# Supplementary material for: Combination of Anti-Angiogenics and Checkpoint Inhibitors for Renal Cell Carcinoma: Is the Whole Greater Than the Sum of Its Parts?
Source: Cancers (Basel). 2022 Jan 27;14(3):644. doi: 10.3390/cancers14030644 (PMC8833428; doi:10.3390/cancers14030644)
Supplement: Supplementary file 1 [file cancers-14-00644-s001.zip › cancers-1546259-supplementary.pdf]

SUPPLEMENTARY MATERIALS

Combination of anti-angiogenics and checkpoint inhibitors for renal cell carcinoma: is the whole greater than the sum of its parts?

Eric Jonasch, Michael B. Atkins, Simon Chowdhury, Paul Mainwaring

Supplementary Table S1. Anti-angiogenic agents and immune checkpoint inhibitors for the treatment of aRCC

(a) PubMed literature search—preclinical data up to July 2020

#	Search terms and inclusion criteria	Results	Topic
1	kidney cancer.ti,ab OR renal cell carcinoma.ti,ab OR renal cell cancer.ti,ab OR rcc.ti,ab OR clear cell renal carcinoma.ti,ab OR clear cell kidney cancer.ti,ab	42460	RCC
2	Cabozantinib.ti,ab OR sunitinib.ti,ab OR pazopanib.ti,ab OR axitinib.ti,ab OR bevacizumab.ti,ab OR tivozanib.ti,ab OR antiangiogenic therapy.ti,ab	24109	Anti-angiogenic agents
3	immune modulation.ti,ab OR immune modulatory.ti,ab OR t cell infiltration.ti,ab OR mdsc.ti,ab OR monocytes.ti,ab OR Immunosuppressive.ti,ab OR dendritic cell.ti,ab OR tumor-infiltrating.ti,ab OR dendritic cells.ti,ab	216734	Immune-modulatory activities
4	1 and 2 and 3	61	
5	Limit to original studies assessing immune-modulatory effects in RCC	10	Screening
6	Manual screen of bibliographic reference lists of studies included in this review	17	Cross-referencing

RCC, renal cell carcinoma.

Preclinical references selected for inclusion

1. Adotevi O, Pere H, Ravel P, et al. A decrease of regulatory T cells correlates with overall survival after sunitinib-based antiangiogenic therapy in metastatic renal cancer patients. *J Immunother* 2010; **33**: 991–8.
2. Alfaro C, Suarez N, Gonzalez A, et al. Influence of bevacizumab, sunitinib and sorafenib as single agents or in combination on the inhibitory effects of VEGF on human dendritic cell differentiation from monocytes. *Br J Cancer* 2009; **100**: 1111–19.
3. Balan M, Mier y Teran E, Waaga-Gasser AM, et al. Novel roles of c-Met in the survival of renal cancer cells through the regulation of HO-1 and PD-L1 expression. *J Biol Chem* 2015; **290**: 8110–20.
4. Garcia JA, Mekhail T, Elson P, et al. Clinical and immunomodulatory effects of bevacizumab and low-dose interleukin-2 in patients with metastatic renal cell carcinoma: results from a phase II trial. *BJU Int* 2011; **107**: 562–70.
5. Guislain A, Gadiot J, Kaiser A, et al. Sunitinib pretreatment improves tumor-infiltrating lymphocyte expansion by reduction in intratumoral content of myeloid-derived suppressor cells in human renal cell carcinoma. *Cancer Immunol Immunother* 2015; **64**: 1241–50.

6. Hara T, Miyake H, Hinata N, Fujisawa M. Inhibition of Tumor Growth and Sensitization to Sunitinib by RNA Interference Targeting Programmed Death-ligand 1 in Mouse Renal Cell Carcinoma RenCa Model. *Anticancer Res*. 2019;39:4737–4742.
7. Hipp MM, Hilf N, Walter S, et al. Sorafenib, but not sunitinib, affects function of dendritic cells and induction of primary immune responses. *Blood* 2008; **111**: 5610–20.
8. Ko JS, Zea AH, Rini BI, et al. Sunitinib mediates reversal of myeloid-derived suppressor cell accumulation in renal cell carcinoma patients. *Clin Cancer Res* 2009; **15**: 2148–57.
9. Kusmartsev S, Eruslanov E, Kubler H, et al. Oxidative stress regulates expression of VEGFR1 in myeloid cells: link to tumor-induced immune suppression in renal cell carcinoma. *J Immunol* 2008; **181**: 346–53.
10. Kwilas AR, Ardiani A, Donahue RN, Aftab DT, Hodge JW. Dual effects of a targeted small-molecule inhibitor (cabozantinib) on immune-mediated killing of tumor cells and immune tumor microenvironment permissiveness when combined with a cancer vaccine. *J Transl Med* 2014; **12**: 294.
11. Li H, Ding J, Lu M, et al. CAIX-specific CAR-T cells and sunitinib show synergistic effects against metastatic renal cancer models. *J Immunother* 2019; doi: 10.1097/CJI.0000000000000301.
12. Liu XD, Hoang A, Zhou L, et al. Resistance to antiangiogenic therapy is associated with an immunosuppressive tumor microenvironment in metastatic renal cell carcinoma. *Cancer Immunol Res* 2015; **3**: 1017–29.
13. Panka DJ, Liu Q, Geissler AK, Mier JW. Effects of HDM2 antagonism on sunitinib resistance, p53 activation, SDF-1 induction, and tumor infiltration by CD11b+/Gr-1+ myeloid derived suppressor cells. *Mol Cancer* 2013; **12**: 17.
14. van Cruijsen H, van der Veldt AA, Vroling L, et al. Sunitinib-induced myeloid lineage redistribution in renal cell cancer patients: CD1c+ dendritic cell frequency predicts progression-free survival. *Clin Cancer Res* 2008; **14**: 5884–92.
15. Wallin JJ, Bendell JC, Funke R, et al. Atezolizumab in combination with bevacizumab enhances antigen-specific T-cell migration in metastatic renal cell carcinoma. *Nat Commun* 2016; **7**: 12624.
16. Yuan H, Cai P, Li Q, et al. Axitinib augments antitumor activity in renal cell carcinoma via STAT3-dependent reversal of myeloid-derived suppressor cell accumulation. *Biomed Pharmacother* 2014; **68**: 751–6.
17. Zhang Q, Tian K, Xu J, et al. Synergistic effects of cabozantinib and EGFR-specific CAR-NK-92 cells in renal cell carcinoma. *J Immunol Res* 2017; **2017**: 6915912.

(b) PubMed literature search—clinical data up to July 2020

#	Search terms and inclusion criteria	Results	Topic
1	kidney cancer.ti,ab OR renal cell carcinoma.ti,ab OR renal cell cancer.ti,ab OR rcc.ti,ab OR clear cell renal carcinoma.ti,ab OR clear cell kidney cancer.ti,ab	42460	RCC
2	Cabozantinib.ti OR sunitinib.ti OR pazopanib.ti OR axitinib.ti OR bevacizumab.ti OR tivozanib.ti OR lenvatinib.ti	12943	Anti-angiogenic agents
3	avelumab.ti OR pembrolizumab.ti OR nivolumab.ti OR avelumab.ti OR ipilimumab.ti OR atezolizumab.ti OR tremelimumab.ti	4688	Checkpoint inhibitors
4	1 and 2 and 3	69	Combination therapy
5	Limit to phase 1, phase 2 or phase 3 comparative trials assessing combinations of a checkpoint inhibitor plus a TKI or an anti-VEGF in patients with advanced renal cell carcinoma.[a]	16	Screening

[a] Screening exclusions: trials in progress; trials reporting quality-of-life and patient-reported outcomes; trials in patients at high risk of relapse after nephrectomy.

RCC, renal cell carcinoma; TKI, tyrosine kinase inhibitor; VEGF, vascular endothelial growth factor.

Clinical references selected for inclusion

1. Atkins MB, Plimack ER, Puzanov I, et al. Axitinib in combination with pembrolizumab in patients with advanced renal cell cancer: a non-randomised, open-label, dose-finding, and dose-expansion phase 1b trial. *Lancet Oncol* 2018; **19**: 405–15.
2. Amin A, Plimack ER, Ernstoff MS, et al. Safety and efficacy of nivolumab in combination with sunitinib or pazopanib in advanced or metastatic renal cell carcinoma: the CheckMate 016 study. *J Immunother Cancer* 2018; **6**: 109.
3. Choueiri TK, Larkin J, Oya M, et al. Preliminary results for avelumab plus axitinib as first-line therapy in patients with advanced clear-cell renal-cell carcinoma (JAVELIN Renal 100): an open-label, dose-finding and dose-expansion, phase 1b trial. *Lancet Oncol* 2018; **19**: 451–60.
4. Choueiri TK, Motzer RJ, Rini BI, et al. Updated efficacy results from the JAVELIN Renal 101 trial: first-line avelumab plus axitinib versus sunitinib in patients with advanced renal cell. *Ann Oncol*. 2020;S0923–7534(20)39308-X.
5. Dudek AZ, Liu LC, Gupta S, et al. Phase Ib/II Clinical Trial of Pembrolizumab With Bevacizumab for Metastatic Renal Cell Carcinoma: BTCRC-GU14-003. *J Clin Oncol*. 2020;38:1138–1145.
6. McDermott DF, Huseni MA, Atkins MB, et al. Clinical activity and molecular correlates of response to atezolizumab alone or in combination with bevacizumab versus sunitinib in renal cell carcinoma. *Nat Med* 2018; **24**: 749–57.
7. McGregor BA, McKay RR, Braun DA, et al. Results of a Multicenter Phase II Study of Atezolizumab and Bevacizumab for Patients With Metastatic Renal Cell Carcinoma With Variant Histology and/or Sarcomatoid Features. *J Clin Oncol*. 2020;38:63–7.
8. Motzer RJ, Penkov K, Haanen J, et al. Avelumab plus axitinib versus sunitinib for advanced renal-cell carcinoma. *N Engl J Med* 2019; **380**: 1103–15.
9. Motzer RJ, Rini BI, McDermott DF, et al. Nivolumab plus ipilimumab versus sunitinib in first-line treatment for advanced renal cell carcinoma: extended follow-up of efficacy and safety results from a randomised, controlled, phase 3 trial. *Lancet Oncol*. 2019;20:1370–1385.
10. Rini BI, Stein M, Shannon P, et al. Phase 1 dose-escalation trial of tremelimumab plus sunitinib in patients with metastatic renal cell carcinoma. *Cancer* 2011; **117**: 758–67.
11. Rini BI, Motzer RJ, Powles T, et al. Atezolizumab plus Bevacizumab Versus Sunitinib for Patients with Untreated Metastatic Renal Cell Carcinoma and Sarcomatoid Features: A Prespecified Subgroup Analysis of the IMmotion151 Clinical Trial. *Eur Urol*. 2020;S0302–2838
12. Rini BI, Plimack ER, Stus V, et al. Pembrolizumab plus axitinib versus sunitinib for advanced renal-cell carcinoma. *N Engl J Med* 2019; **380**: 1116–27.
13. Rini BI, Powles T, Atkins MB, et al. Atezolizumab plus bevacizumab versus sunitinib in patients with previously untreated metastatic renal cell carcinoma (IMmotion151): a multicentre, open-label, phase 3, randomised controlled trial. *Lancet* 2019; **393**: 2404–15.
14. Taylor MH, Lee CH, Makker V, et al. Phase IB/II Trial of Lenvatinib Plus Pembrolizumab in Patients With Advanced Renal Cell Carcinoma, Endometrial Cancer, and Other Selected Advanced Solid Tumors. *J Clin Oncol*. 2020;38:1154–1163.
15. Uemura M, Tomita Y, Miyake H, et al. Avelumab plus axitinib vs sunitinib for advanced renal cell carcinoma: Japanese subgroup analysis from JAVELIN Renal 101. *Cancer Sci*. 2020;111:907–923.
16. Wallin JJ, Bendell JC, Funke R, et al. Atezolizumab in combination with bevacizumab enhances antigen-specific T-cell migration in metastatic renal cell carcinoma. *Nat Commun* 2016; **7**: 12624.

(c) Congress abstracts

Manual searches were conducted using the proceedings from key congresses (2016, 2017, 2018, 2019, 2020), considered by the authors as being most relevant (the Annual Meeting of the American Society of Clinical Oncology [ASCO], the ASCO Genitourinary Cancers Symposium [ASCO GU]; the Annual Meeting of the European Society for Medical Oncology [ESMO]). Search terms and inclusion criteria: phase 1, phase 2, or phase 3 comparative trials assessing combinations of a checkpoint inhibitor plus a tyrosine kinase inhibitor or an anti-vascular endothelial growth factor antibody in patients with advanced renal cell carcinoma.

Abstract references selected for inclusion

1. Agarwal N, Green M, di Nucci F, et al. Phase Ib study (COSMIC-021) of cabozantinib in combination with atezolizumab: results of the dose escalation stage in patients (pts) with treatment-naïve advanced renal cell carcinoma (RCC). *Ann Oncol* 2018: 29 (suppl. 8): viii303–viii331.
2. Apolo AB, Mortazavi A, Hu ZI, et al. Circulating tumor cell (CTC) enumeration in patients (pts) with metastatic genitourinary (mGU) tumors treated in a phase I study of cabozantinib and nivolumab (CaboNivo) +/- ipilimumab (CaboNivoIpi). *J Clin Oncol* 2019; 37 (15 Suppl): 4555–4555.
3. Barthelemy P, Escudier B, Negrier S, et al. TiNivo: Tivozanib combined with nivolumab results in prolonged progression free survival in patients with metastatic renal cell carcinoma (mRCC): Final results. *Annals Oncol* 2019:30 (suppl_5): v356–v402. 10.1093/annonc/mdz249
4. Choueiri TK, Larkin JMG, Pal SK, et al. Efficacy and biomarker analysis of patients (pts) with advanced renal cell carcinoma (aRCC) with sarcomatoid histology (sRCC): Subgroup analysis from the phase III JAVELIN renal 101 trial of first-line avelumab plus axitinib (A 1 Ax) vs sunitinib (S). *Annals Oncol* 2019:30 (suppl_5): v356–v402. 10.1093/annonc/mdz249
5. Choueiri TK, Albiges L, Powels T, et al. A phase III study (COSMIC-313) of cabozantinib in combination with nivolumab and ipilimumab in patients with previously untreated advanced renal cell carcinoma of intermediate or poor-risk. *J Clin Oncol* 2020; 38 (suppl): TPS5102.
6. Chowdhury S, McDermott DF, Henner Voss M, et al. A phase I/II study to assess the safety and efficacy of pazopanib and pembrolizumab in patients with advanced renal cell carcinoma. *J Clinl Oncol* 2017; 35 (15 suppl.): 4506.
7. Dudek AZ, Sidani A, Gopalji G, et al. Phase Ib study of pembrolizumab in combination with bevacizumab for the treatment of metastatic renal cell carcinoma: Big Ten Cancer Research Consortium BTCRC-GU14-003. *J Clin Oncol* 2016; 34 (2 suppl.): 559.
8. Escudier BB, Ravaud, A, Negrier S, et al. Tivozanib combined with nivolumab: phase Ib/II study in metastatic renal cell carcinoma (mRCC). *J Clin Oncol* 2018; 36 (6 suppl.): 618.
9. Filippot R, McGregor BA, Flaifel A, et al. Atezolizumab plus bevacizumab in non-clear cell renal cell carcinoma (NccRCC) and clear cell renal cell carcinoma with sarcomatoid differentiation (ccRCCsd): Updated results of activity and predictive biomarkers from a phase II study. *J Clin Oncol* 2019; 37 (15 Suppl): 4583–4583.
10. Gao J, Karam JA, Tannir NM, et al. A pilot randomized study evaluating nivolumab (nivo) or nivo + bevacizumab (bev) or nivo + ipilimumab (ipi) in patients with metastatic renal cell carcinoma (MRCC) eligible for cytoreductive nephrectomy (CN), metastasectomy (MS) or post-treatment biopsy (Bx). *J Clin Oncol* 2019; 37 (15 Suppl): 4520–4520.
11. Grünwald V, Grüllich C, Ivanyi P, et al. A phase II trial of TKI induction followed by a randomized comparison between nivolumab or TKI continuation in renal cell carcinoma (NIVOSWITCH). *Annals Oncol* 2019:30 (suppl_5): v388.
12. Keeler ME, Bernard B, Weisdack S, et al. Pembrolizumab (pembro) and cabozantinib (cabo) in patients (pts) with metastatic renal cell carcinoma (mRCC): phase I results. *J Clin Oncol* 2019; 37 (7 suppl.): 600.
13. Lee CH MV, Rasco DW, Taylor MH, et al. Lenvatinib + pembrolizumab in patients with renal cell carcinoma: updated results. *J Clin Oncol* 2018; 36 (6 suppl.): 4560.

14. Lee C-H SA, Hsieh JJ, Rao A, et al. Phase II trial of lenvatinib (LEN) plus pembrolizumab (PEMBRO) for disease progression after PD-1/PD-L1 immune checkpoint inhibitor (ICI) in metastatic clear cell renal cell carcinoma (mccRCC). *J Clin Oncol 2020*; 38 (suppl): abstr 5008.
15. Nadal RM, Stein, M, Sumanta K, et al. Results of phase I plus expansion cohorts of cabozantinib (Cabo) plus nivolumab (Nivo) and CaboNivo plus ipilimumab (Ipi) in patients (pts) with with metastatic urothelial carcinoma (mUC) and other genitourinary (GU) malignancies. *J Clin Oncol* 2018; 36 (6 suppl.): 515.
16. Plimack ER, Rini BI, Stus V, et al. Pembrolizumab plus axitinib versus sunitinib as first-line therapy for advanced renal cell carcinoma (RCC): Updated analysis of KEYNOTE-426. *J Clin Oncol*. 2020;38(suppl):abstr 5001.
17. Rini BI, Motzer RJ, Powles T, et al. Atezolizumab (atezo) + bevacizumab (bev) versus sunitinib (sun) in pts with untreated metastatic renal cell carcinoma (mRCC) and sarcomatoid (sarc) histology: IMmotion151 subgroup analysis. *J Clin Oncol* 2019; 37 (15 Suppl): 4512–4512.[a]
18. Rini BI, Plimack ER, Stus V, et al. Pembrolizumab (pembro) plus axitinib (axi) versus sunitinib as first-line therapy for metastatic renal cell carcinoma (mRCC): Outcomes in the combined IMDC intermediate/poor risk and sarcomatoid subgroups of the phase 3 KEYNOTE-426 study. *J Clin Oncol* 2019; 37 (15 Suppl): 4500–4500.
19. Uemura M, Tomita Y, Miyake H, et al. Randomized phase III trial of avelumab 1 axitinib vs sunitinib as firstline treatment for advanced renal cell carcinoma: JAVELIN renal 101 Japanese subgroup analysis. Annals Oncol 2019:30 (suppl_5): v356–v402. 10.1093/annonc/mdz249[a]
20. Zhang T, Ballman KV, Choudhury AD, et al. PDIGREE: An adaptive phase 3 trial of PD-inhibitor nivolumab and ipilimumab (IPI-NIVO) with VEGF TKI cabozantinib (CABO) in metastatic untreated renal cell cancer (Alliance A031704) *J Clin Oncol* 2019; 37 (15 Suppl): DOI: 10.1200/JCO.2019.37.15_suppl.TPS4596.
21. Zhang T, Ballman KV, Choudhury AD, et al. PDIGREE: An adaptive phase III trial of PD-inhibitor nivolumab and ipilimumab (IPI-NIVO) with VEGF TKI cabozantinib (CABO) in metastatic untreated renal cell cancer (Alliance A031704) *J Clin Oncol 2020*; 38 (suppl): TPS5100.
22. Zibelman MR, Geynisman DM, Molina AM, et al. Phase I/II study of axitinib (axi) and nivolumab (nivo) in patients with metastatic renal cell carcinoma (mRCC). *J Clin Oncol* 2019; 37 (15 Suppl): 4567–4567

[a]Final publication identified by clinical trial searches

Supplementary Table S2. Anti-angiogenic agents and immune checkpoint inhibitors for the treatment of aRCC

Agent	Target(s)	Type of agent (status)
**Anti-angiogenic agents (inhibitors of the VEGF pathway)**		
Sorafenib	VEGFR, Raf	TKI (licensed)
Pazopanib	VEGFR, PDGFR, FGFR, c-Kit	TKI (licensed)
Sunitinib	VEGFR, PDGFR, c-Kit, FLT3	TKI (licensed)
Axitinib	VEGFR, PDGFR, c-Kit	TKI (licensed)
Cabozantinib	VEGFR, MET, AXL, RET, c-Kit, FLT3, TRKB, Tie-2	TKI (licensed)
Lenvatinib	VEGFR, PDGFR, FGFR	TKI (licensed)
Tivozanib	VEGFR	TKI (licensed)
Bevacizumab	VEGF	Monoclonal antibody (licensed)
DC101	VEGFR	Monoclonal antibody (investigational)
**Immune checkpoint inhibitors**		
Nivolumab	PD-1	Monoclonal antibody (licensed)
Pembrolizumab	PD-1	Monoclonal antibody(licensed)
Toripalimab	PD-1	Monoclonal antibody (licensed)
Atezolizumab	PD-L1	Monoclonal antibody (licensed)
Avelumab	PD-L1	Monoclonal antibody (licensed)
Durvalumab	PD-L1	Monoclonal antibody (licensed)
Ipilimumab	CTLA-4	Monoclonal antibody (licensed)
Tremelimumab	CTLA-4	Monoclonal antibody (licensed)

aRCC, advanced renal cell carcinoma; CTLA-4, cytotoxic T lymphocyte antigen 4; FGFR, fibroblast growth factor receptor; FLT-3, FMS-like tyrosine kinase 3; MET, mesenchymal-epithelial transition factor or hepatocyte growth factor receptor; PD-1, programmed cell death protein 1; PDGFR, platelet-derived growth factor receptor; PD-L1, programmed cell death ligand 1; RET, rearranged during transfection receptor; Tie-2, tyrosine kinase with immunoglobulin and epidermal growth factor homology domains-2; TKI, tyrosine kinase inhibitor; VEGF, vascular endothelial growth factor; VEGFR, vascular endothelial growth factor receptor.

Supplementary Table S3. Considerations for interpreting results of CPI/ anti-VEGF treatment combinations in RCC[a]

Consideration	Issue(s)		Comments/implications
Patient population	Between-trial differences in:	IMDC risk profile[b]	• This may impact response to treatment combination:<br>– In favour of CPI combination in intermediate/poor risk patients<br>– In favour of sunitinib in patients with favourable risk
		Tumour PD-L1 expression level/threshold/subgroup sizes	• Assays differ between trials. No gold standard for PD-L1 expression<br>• PD-L1 expression is associated with intermediate/poor risk disease; greater benefit with CPI combination and less benefit for TKI<br>• Implications of PD-L1 expression for TKI–CPI combination remain unclear
Trial design	Differences in trial protocols and trial design		• Trial outcomes differ:<br>– PFS (*vs* IrPFS), ORR (*vs* IrORR), investigator- or IRC-defined outcomes (vs than externally validated definitions)<br>– Some trials permit (*vs* do not) treatment beyond progression<br>– No trials have used landmark PFS or OS or treatment-free survival<br>• Approaches to treatment discontinuation and censoring differ<br>• Study design can differ:<br>– Use of unblinded and crossover designs<br>– Dates and locations of trials will affect availability of salvage (CPI or other) therapies and relative incentive to switch
Length of accrual and analysis timing	Potential to compromise OS result in sequential TKI–CPI arm		• Patients with the most aggressive disease allocated to TKI trial arms may not have had access to second-line CPI therapy resulting in earlier mortality and early distortion of the OS hazard ratio in favour of CPI-containing arm
Safety assessment and toxicity management	TKI target and pharmacokinetics		• Different receptor tyrosine kinases targeted by TKIs may influence their respective safety profiles<br>• Different TKI half-lives may lead to challenges in assigning treatment-related AEs particularly for those potentially common to TKI and CPIs (e.g. diarrhoea, liver function test abnormalities)
	Use of QoL assessment tool and schedule		• QoL will vary across the treatment cycle (e.g. worse during sunitinib treatment than during the 2-week break); outcomes will be influenced by the timing of assessment<br>• Censoring (or not) from the analysis of patients off CPI therapy due to toxicity will affect the outcome:<br>– Some patients may have persistent toxicity and impaired QoL<br>– Some patients may find toxicities improve and may experience sustained response with improved QoL

[a]Based on findings from pivotal benchmark CPI-based combination trials using sunitinib as the control arm. [b]Current studies were not powered to assess outcome as an assessment of risk. AE, adverse event; CPI, immune checkpoint inhibitors; IMDC, International Metastatic RCC Database Consortium; IRC, independent review committee; IrPFS, immune-related progression-free survival; ORR, objective response rate; OS, overall survival; PD-L1, programmed cell death ligand 1; PFS, progression-free survival; QoL, quality of life; RCC, renal cell carcinoma; TKI, tyrosine kinase inhibitor; VEGFR; vascular endothelial growth factor.

Supplementary Figure S1. Schematic diagram of the possible mechanisms of action of anti-angiogenic plus CPI combination therapy in RCC

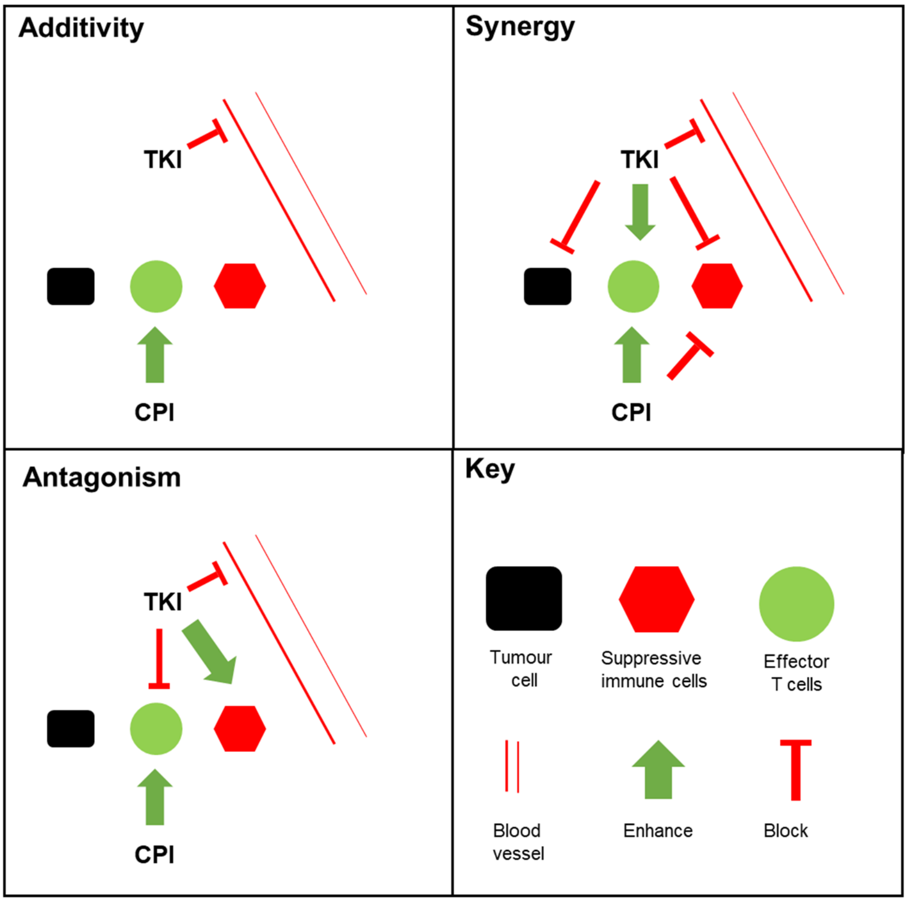

CPI, checkpoint inhibitor; RCC, renal cell carcinoma; TKI, tyrosine kinse inhibitor
